# The Medico-Psychological Society

**Published:** 1897-08-28

**Authors:** 


### THE MEDICO-PSYCHOLOGICAL SOCIETY.

We reprint a letter which appeared in the *British Medical Journal* of August 21st on the subject of the true value as a test of efficiency represented by the certificate of the above society:—

"Sir,—In reading asylum reports for the past year one is struck by the number of candidates who are said to have passed the above examination and by the remarks attesting to the enormous value such an examination has in perfecting the nursing of the insane.

"Of late it has become the common practice of asylum medical officers to declare the superiority of the holders of certificates for the above examination over trained hospital nurses, as they have, in addition to ordinary sick nursing, experience in nursing the insane, and it is only just that medical men generally should know what is the real value of the certificate awarded for the above examination.

"First, as regards the class of candidates. The great majority of asylum nurses are drawn from the ranks of domestic servants, and although most of them have had a fair elementary education, yet, even in these days, a large proportion of them can only write and spell imperfectly. So much for the raw material.

"As regards the course of study, there is a book called 'Handbook for Attendants on the Insane.' It consists of 117 pages, and is the joint effort of eleven alienists. The contents of this book are required to be mastered in two years with the aid of lectures by the medical staff of asylums.

"The examination consists of written and oral parts. The paper for the written part is set by examiners appointed by the Medico-Psychological Association; the *vivâ voce* part is conducted by the superintendent of the particular asylum in which the attendants are engaged, with the help of an assessor, who is generally a friendly superintendent; they also mark the papers. As the number of successful candidates redounds to the credit of the superintendent, this part of the examination can scarcely be an impartial one.

"As to the standard of work required: if the official text-book is perused it will be seen that it contains a very superficial smattering of anatomy and physiology, with thirteen pages devoted to symptoms of disease and disorder, fifteen to mind and its disorder, nineteen to sick nursing, and thirteen to mental nursing. Taking the standard of work required from the official handbook, together with the method of examination, one cannot but think that the value of the certificate is not what its high-sounding name leads one to expect.

"The rarity of unsuccessful candidates is a curious phenomenon about this examination. In all examinations which have any pretensions to any standard at all, there is always a percentage of failures, yet in this examination it is no uncommon thing for batches of attendants in various asylums to all present themselves successfully.

"A guide to the standard and value of the examination may be taken from the examination of the St. John Ambulance Association in first aid. It frequently happens that candidates for the Medico-Psychological Association's examination are prepared simultaneously for the First Aid Examination of the St. John Ambulance Association, and, whereas, the successful candidates for the former examination are often 100 per cent., yet the same candidates for the latter supply a fair amount of failures, although it is an examination which is easily passed by policemen and railway porters of average intelligence.

"I do not wish to decry in any sense the systematic training of asylum attendants by means of lectures and examinations, but, when the Medico-Psychological Association takes upon itself the business of launching into the world certificated individuals, surely the general medical world and the public may expect that such certificates should indicate that the holders possess that sound knowledge and ability which has enabled them to pass an examination which should be thorough and impartial.

"No objection can be urged against superintendents of asylums training and examining their own attendants and nurses, and using their own certificates, as is the practice in hospitals, but it is certainly harmful for a public body to organise a system of training and examination of an exceedingly low standard, and after delegating the functions of examiners to individuals who cannot be impartial, and who are perhaps even unconnected with them, to issue certificates which are really no guarantee of knowledge or proficiency.

"I may say that I speak from personal experience, having for some years prepared candidates for the above examinations with what must have been flattering success were not the real facts of the case known to me.—I am, &c.,

"August 16th." "M. P. C.

